# Diffuse Nephromegaly With Dialysis‐Dependent Acute Kidney Injury as the Initial Presentation of Primary Mediastinal Large B‐Cell Lymphoma

**DOI:** 10.1155/crin/4177843

**Published:** 2026-11-04

**Authors:** Zhong Xhen Khor

**Affiliations:** ^1^ Department of Internal Medicine IMU University Seremban Negeri Sembilan Malaysia imu.edu.my

**Keywords:** acute kidney injury, HIV, lymphomatous kidney infiltration, nephromegaly, primary mediastinal large B-cell lymphoma

## Abstract

Diffuse renal enlargement is an uncommon manifestation of lymphoma and may mimic primary renal disorders. We report a young woman who presented with acute kidney injury (AKI) and bilateral ballotable flank masses, initially suspected to represent polycystic kidney disease. Chest radiography demonstrated a large mediastinal mass, prompting cross‐sectional imaging that revealed bulky anterior mediastinal disease with widespread nodal and extranodal involvement. Imaging showed asymmetric bilateral renal enlargement, markedly greater on the left, with diffuse hypodense infiltrative lesions radiologically suspicious for lymphomatous involvement. Core biopsy of the mediastinal mass confirmed primary mediastinal large B‐cell lymphoma (PMBCL), and concurrent testing identified newly diagnosed human immunodeficiency virus infection. The patient developed anuric, dialysis‐dependent AKI and died before definitive rituximab‐based chemotherapy could be initiated. This case highlights an atypical and aggressive presentation of PMBCL with presumed diffuse renal involvement, emphasizing the need to consider infiltrative malignancy in patients with unexplained nephromegaly and AKI.

## 1. Introduction

Primary mediastinal large B‐cell lymphoma (PMBCL) is a distinct clinicopathological entity within aggressive large B‐cell lymphomas that predominantly affects adolescents and young adults and usually presents as a bulky anterior mediastinal mass. At diagnosis, disease is commonly thorax predominant, whereas extensive extranodal dissemination is less typical [1–3].

Renal involvement in lymphoma is well recognized but is frequently clinically silent. When clinically apparent, it may occur as multiple renal masses, direct extension from adjacent disease, solitary lesions, or diffuse interstitial infiltration; the latter may produce renal enlargement and acute kidney injury (AKI) [4–6]. These appearances can mimic primary renal disease and complicate attribution of kidney injury when systemic lymphoma has not yet been recognized.

Human immunodeficiency virus (HIV) infection is clinically relevant because HIV‐associated lymphomas may present with advanced and extranodal disease [7]. We report a young woman with PMBCL and presumed diffuse lymphomatous renal involvement presenting with rapidly progressive, dialysis‐dependent AKI and asymmetric renal enlargement. The case highlights an uncommon renal‐dominant presentation of PMBCL and the diagnostic challenge of distinguishing malignant renal infiltration from other causes of renal enlargement and AKI.

## 2. Case Presentation

A previously well woman in her early 20s presented with a 1‐month history of nonspecific abdominal discomfort. Initial evaluation in primary care revealed an elevated serum creatinine of 320 μmol/L, prompting referral for suspected chronic kidney disease. There was no history of trauma, nephrotoxic drug use, or family history of kidney disease.

On admission, she was tachycardic at 124 beats/min but remained normotensive (100/64 mmHg), with an oxygen saturation of 95% on room air and no clinical features of shock. She appeared mildly dyspneic. Cardiovascular and respiratory examinations were unremarkable. Abdominal examination revealed bilateral large, ballotable flank masses, with no peripheral lymphadenopathy, hepatomegaly, or splenomegaly. She, otherwise, remained normotensive throughout admission, albeit with baseline tachycardia.

Point‐of‐care ultrasonography demonstrated bilaterally enlarged kidneys, initially raising suspicion for autosomal dominant polycystic kidney disease. However, chest radiography revealed a large mediastinal mass (Figure 1a), highlighting a discordance between abdominal findings and thoracic imaging.

**FIGURE 1:**
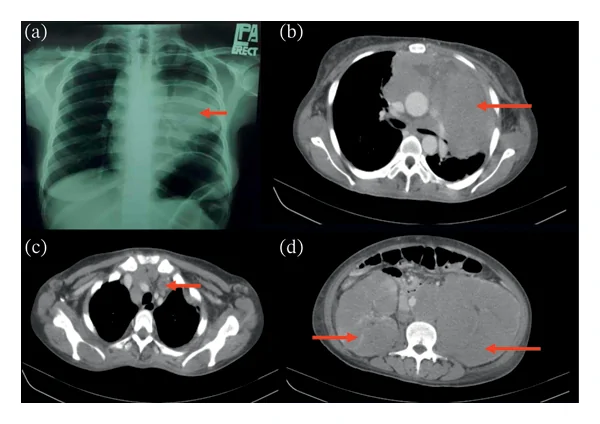
Radiological features of primary mediastinal large B‐cell lymphoma with presumed diffuse renal involvement. (a) Chest radiograph demonstrating a large left mediastinal opacity with widening of the mediastinum, raising suspicion for an underlying mediastinal mass (red arrow). (b) Axial contrast‐enhanced CT thorax showing a bulky anterior mediastinal mass (red arrow) occupying the left hemithorax and compressing adjacent mediastinal structures. (c) Axial contrast‐enhanced CT thorax demonstrating left brachiocephalic venous thrombosis/filling defect (red arrow) associated with mediastinal mass effect. (d) Axial contrast‐enhanced CT abdomen showing asymmetric bilateral renal enlargement, markedly greater on the left (red arrows), with diffuse hypodense infiltrative renal parenchymal lesions radiologically suspicious for lymphomatous involvement.

Despite this development, the patient requested discharge against medical advice. Two weeks later, she represented with worsening lethargy, anuria, and rapidly progressive renal impairment, with serum creatinine rising to approximately 1000 μmol/L. She required urgent initiation of hemodialysis. Lab results are summarized in Table 1.

**TABLE 1:** Selected renal and metabolic investigations during initial presentation and representation with severe acute kidney injury.

Laboratory parameter	Initial admission	Representation with severe AKI
Sodium (mmol/L)	132	125
Potassium (mmol/L)	4.62	5.16
Urea (mmol/L)	9.4	25.2
Creatinine (μmol/L)	372	1038
Lactate dehydrogenase (U/L)	1429	1615
Uric acid (μmol/L)	862	1114
Calcium (mmol/L)	2.56	2.21
Phosphate (mmol/L)	1.80	3.30

*Note:* Urinalysis, quantitative proteinuria, and serum bicarbonate were not available in the laboratory dataset provided.

Abbreviation: AKI, acute kidney injury.

Formal abdominal ultrasonography demonstrated enlargement of both kidneys with increased echogenicity and reduced corticomedullary differentiation bilaterally, more pronounced on the left. There was asymmetric bilateral renal enlargement, markedly greater on the left, with the left kidney measuring 18 cm and the right 12.4 cm. There was no hydronephrosis or renal calculus. Multiple enlarged intra‐abdominal lymph nodes, some matted within the left pararenal and pelvic regions, were identified with minimal free fluid. These findings raised suspicion of an infiltrative or lymphoproliferative process, prompting cross‐sectional imaging.

Contrast‐enhanced computed tomography of thorax, abdomen, and pelvis revealed a large anterior mediastinal mass measuring 9.4 × 13.6 × 9.6 cm, encasing the pulmonary artery and compressing adjacent bronchi (Figure 1b). There was extensive nodal disease involving regions above and below the diaphragm. Additional findings included multiple bilateral pulmonary nodules with surrounding ground‐glass halo. A left brachiocephalic vein thrombosis was identified (Figure 1c), along with moderate pericardial and pleural effusions. The overall imaging appearances were consistent with disseminated lymphoproliferative disease.

Notably, the kidneys were asymmetrically enlarged, markedly greater on the left (18 cm versus 12.4 cm). The renal parenchyma was diffusely replaced by confluent hypodense infiltrative lesions, radiologically suspicious for lymphomatous involvement (Figure 1d).

Core biopsy of the mediastinal mass demonstrated fibrocollagenous tissue diffusely infiltrated by medium‐to‐large malignant lymphoid cells with compartmentalizing fibrosis (Figure 2a). Immunohistochemistry showed diffuse CD20 (Figure 2b), MUM1 expression (Figure 2c), PAX5 positivity (Figure 2d), and BCL2 and BCL6 expressions. There was weak CD30 positivity and no expression of CD3, CD5, CD10, CD117, or pancytokeratin. The Ki‐67 proliferative index was greater than 85%, supporting a diagnosis of PMBCL. Concurrent HIV testing confirmed newly diagnosed HIV infection, with a plasma HIV RNA level of 20,721 copies/mL. CD4 and CD8 lymphocyte counts were unavailable because the specimen could not be processed following collection in an incorrect tube.

**FIGURE 2:**
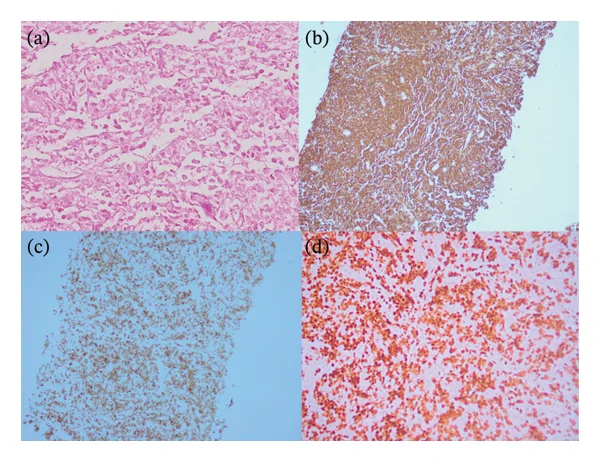
Histopathological and immunohistochemical features of the mediastinal core biopsy. (a) High‐power hematoxylin and eosin section demonstrating medium‐to‐large atypical lymphoid cells with irregular nuclear contours and scant‐to‐moderate cytoplasm. (b) Immunohistochemistry showing diffuse CD20 positivity in the neoplastic cells. (c) Immunohistochemistry showing diffuse MUM1 expression. (d) Immunohistochemistry showing diffuse PAX5 positivity, supporting B‐cell lineage. Together with BCL2 and BCL6 expressions, weak CD30 positivity, and a Ki‐67 proliferative index greater than 85%, these findings supported primary mediastinal large B‐cell lymphoma.

Bridging therapy with intravenous corticosteroids (intravenous dexamethasone 4 mg twice daily) was initiated to reduce tumor burden. Anticoagulation with low molecular weight heparin (subcutaneous enoxaparin 40 mg once daily) was started for left brachiocephalic vein thrombosis, with dose adjustment for renal impairment. Following multidisciplinary discussion with infectious diseases specialists, the initiation of antiretroviral therapy was planned, with consideration of renal impairment. An antiretroviral regimen comprising abacavir, lamivudine, and efavirenz was selected with consideration of her renal impairment.

The patient was referred to a tertiary center for definitive oncological management, including consideration of rituximab‐based chemotherapy. However, treatment planning was complicated by advanced‐stage disease, dialysis‐dependent renal failure, and the need to coordinate chemotherapy with antiretroviral therapy. Despite supportive management, her clinical condition deteriorated rapidly with progression to multiorgan failure, and she subsequently died.

## 3. Discussion

This case represents an unusual renal‐dominant presentation of PMBCL. PMBCL classically produces a bulky anterior mediastinal mass, and pleural or pericardial effusions and venous obstruction may accompany local mass effect [1–3]. In contrast, extensive extranodal disease at initial presentation is less typical. The combination in our patient of biopsy‐proven mediastinal PMBCL, disseminated nodal and extranodal disease, asymmetric renal enlargement, and anuric dialysis‐dependent AKI, therefore, represents an atypical phenotype.

Renal lymphoma is more often secondary to systemic lymphoma than primary kidney‐limited disease. Secondary involvement may occur by hematogenous dissemination or direct extension and can manifest as multiple masses, diffuse infiltration, or renal enlargement [4, 5]. Direct renal infiltration can impair kidney function even when radiological abnormalities are subtle [4]. Diffuse renal enlargement also overlaps with nonmalignant renal conditions, including HIV‐associated nephropathy, which may present with rapidly progressive renal dysfunction, proteinuria, and enlarged echogenic kidneys [8]. In this patient, the confluent hypodense parenchymal lesions and disseminated lymphoma favored presumed secondary lymphomatous involvement, but the absence of renal biopsy prevents histological confirmation.

The AKI was likely multifactorial. Cancer‐associated AKI may result from hemodynamic factors, obstruction, tumor lysis, nephrotoxicity, or direct parenchymal infiltration [6]. Presumed lymphomatous infiltration was considered the dominant mechanism because of the marked renal enlargement and diffuse infiltrative lesions, while obstruction and documented nephrotoxic exposure were absent. HIV‐associated nephropathy could not be fully assessed because urinalysis and quantitative proteinuria were unavailable in the retrievable records. In addition, marked hyperuricemia, hyperphosphatemia, and elevated lactate dehydrogenase were present before definitive cytotoxic chemotherapy, raising the possibility that spontaneous tumor lysis contributed to the renal dysfunction. Conversely, established severe AKI itself may increase uric acid and phosphate concentrations; spontaneous tumor lysis, therefore, remains a possible contributor rather than a proven diagnosis.

Renal biopsy is the definitive method for confirming lymphomatous infiltration and may identify competing renal lesions, but it is not invariably pursued when systemic lymphoma has already been established from a safer, more accessible site and renal histology is unlikely to alter immediate management [4, 5]. In this patient, mediastinal biopsy established PMBCL and the renal imaging was strongly suggestive of secondary involvement. The absence of kidney histology nevertheless remains an important limitation; accordingly, the renal process is described as presumed or radiologically suspected lymphomatous involvement.

Published cases of lymphoma presenting with renal enlargement and AKI have predominantly involved conventional diffuse large B‐cell lymphoma (DLBCL) or lymphoblastic lymphoma rather than PMBCL (Table 2) [9–21]. Several were biopsy confirmed, and the renal course ranged from complete recovery to persistent dialysis dependence. Cetin et al. described biopsy‐confirmed secondary renal DLBCL with AKI requiring hemodialysis; despite hematologic remission, the patient remained dialysis dependent [20]. Bertacchi et al. reported bilateral primary renal DLBCL causing severe kidney failure, with partial renal recovery after chemotherapy and eventual discontinuation of hemodialysis [19]. These reports illustrate that severe AKI may occur in both primary and secondary renal lymphomas. Our case differs in that the underlying lymphoma was PMBCL and the nephrology‐dominant presentation preceded definitive lymphoma‐directed treatment. We did not identify a comparable published PMBCL presentation with this combination of presumed diffuse renal involvement, marked renal enlargement, anuria, and dialysis‐dependent AKI [1–3, 9–21].

**TABLE 2:** Reported cases of lymphoma presenting with bilateral nephromegaly and acute kidney injury.

Year	Author	Lymphoma type	Kidney biopsy	Nephromegaly	AKI (peak creatinine)	HD required	Kidney recovery	Chemotherapy	Regimen	Outcome
2004	Sellin et al.	DLBCL	Yes (renal)	Yes	Yes (391 μmol/L)	No	Complete	Yes	CHOP	Alive (improved)
2009	Monfared et al.	DLBCL	Yes (renal)	Yes	Yes (5.5 mg/dL)	No	None	Yes	CHOP + rituximab	Died
2010	Cohen et al. (index case)	NHL	Yes (renal)	Yes	Yes (5 mg/dL)	NR	Yes (Not specified)	Yes	Rituximab based therapy	Improved; details not specified
2015	Prabhakar et al.	Primary renal lymphoma (DLBCL)	Yes (renal)	Yes	Yes (8.5 mg/dL)	No	Complete	Yes	R‐CHOP	Alive (improved)
2016	Mitome et al.	DLBCL	Yes (renal)	Yes	Yes (2.17 mg/dL)	Yes	Complete	Yes	R‐CHOP	Alive (improved)
2016	Rajakumar et al.	Lymphoblastic lymphoma	Yes (renal)	Yes	Yes (NR)	NR	Partial	Yes	Multiagent	Alive
2016	Saadat et al.	DLBCL	No (extrarenal)	Yes	1.6 mg/dL	No	No	Yes	Rituximab based	Died
2017	Okubo et al.	DLBCL	Yes (renal)	Yes	Yes (Cr 9.01 mg/dL)	Yes (CHDF ⟶ HD)	Complete (initial recovery)	Yes	R‐CHOP–based	Died following CNS relapse
2019	Aminde et al.	Aggressive NHL	No (extrarenal)	Yes	Yes (4.35 mg/dL)	NR	None	No	—	Died
2023	Bai et al. (Case 1)	DLBCL	No (extrarenal)	Yes	Yes (1097 μmol/L)	Yes	Partial	No	—	Died
2023	Bai et al. (Case 2)	DLBCL	Yes (renal)	Yes	Yes (419 μmol/L)	No	Partial	Yes	R‐CHOP	Died
2023	Bai et al. (Case 3)	T‐lymphoblastic lymphoma	Yes (renal)	Yes	Yes (541 μmol/L)	No	Complete	Yes	Hyper‐CVAD	Alive
2025	Bertacchi et al.	Primary renal lymphoma (DLBCL)	Yes (renal)	Yes	Yes (756 μmol/L)	Yes	Partial	Yes	Rituximab‐based multiagent chemotherapy, dose‐adjusted for severe kidney failure	Complete lymphoma remission; HD discontinued after 6 months; eGFR 28 mL/min/1.73 m^2^ at 12 months
2025	Cetin et al.	DLBCL, secondary renal involvement	Yes (renal)	Yes	Cr 4.64 mg/dL	Yes	None	Yes	Da‐R‐CHOEP	Hematological remission; remained dialysis dependent
2025	Darwish and Lightle	T‐lymphoblastic lymphoma	Yes (renal)	Yes	Yes (8.24 mg/dL)	Yes	Partial	Yes	Multiagent	Alive (improved)

*Note:* HD: hemodialysis; CHDF: continuous hemodiafiltration; Da‐R‐CHOEP: daratumumab, rituximab, cyclophosphamide, doxorubicin, vincristine, etoposide, and prednisone. Nephromegaly was defined as bilateral renal enlargement on imaging. “Kidney biopsy” refers specifically to renal tissue biopsy unless, otherwise, stated.

Abbreviations: AKI, acute kidney injury; DLBCL, diffuse large B‐cell lymphoma; eGFR, estimated glomerular filtration rate; NHL, non‐Hodgkin lymphoma; NR, not reported.

The newly diagnosed HIV infection adds clinical complexity but should not be overinterpreted as the cause of the unusual dissemination pattern. HIV‐associated lymphomas may present with aggressive, advanced, and extranodal disease [7], and antiretroviral therapy should be initiated when clinically feasible [22]. Available pooled data also support rituximab‐containing lymphoma therapy in people with HIV when, otherwise, appropriate [23]. In this patient, however, severe organ dysfunction and rapid clinical deterioration precluded definitive rituximab‐based chemotherapy. The extent to which HIV contributed to the pattern of dissemination therefore cannot be determined from a single case.

Renal function may improve with successful lymphoma‐directed therapy, particularly when AKI is driven by reversible infiltrative disease, but outcomes are variable [9, 12, 13, 16, 18–21]. In our patient, death before definitive chemotherapy prevented assessment of reversibility. The principal clinical lesson is, therefore, diagnostic: unexplained renal enlargement with rapidly progressive AKI should prompt consideration of infiltrative malignancy, especially when systemic features are present. In PMBCL, renal involvement is not a typical presenting feature; when encountered, presumed lymphomatous infiltration should be distinguished explicitly from HIV‐related kidney disease, tumor lysis, and other potential contributors to AKI.

## Funding

No funding was received for this work.

## Consent

Publication approval was granted by the Director General, Ministry of Health Malaysia, through the National Institutes of Health Malaysia (Approval reference: NIH.800‐4/4/1 Jld. 164(54)). Written informed consent could not be obtained as the patient was deceased and the next of kin could not be contacted despite reasonable attempts. All efforts have been made to ensure complete anonymization, and no identifiable personal information is included in this report. This circumstance is explained in the cover letter in accordance with the journal’s instructions for authors.

## Conflicts of Interest

The author declares no conflicts of interest.

## Data Availability

Data sharing is not applicable to this article as no datasets were generated or analyzed during the current study.

## References

[1] Giulino-Roth L. , How I Treat Primary Mediastinal B-Cell Lymphoma, Blood. (2018) 132, no. 8, 782–790, 10.1182/blood-2018-04-791566. PMC6634954 29976557

[2] Dunleavy K. and Wilson W. H. , Primary Mediastinal B-Cell Lymphoma and Mediastinal Gray Zone Lymphoma: Do They Require a Unique Therapeutic Approach?, Blood. (2015) 125, no. 1, 33–39, 10.1182/blood-2014-05-575092. PMC4281829 25499450

[3] Pfau D. , Smith D. A. , Beck R. et al., Primary Mediastinal Large B-Cell Lymphoma: A Review for Radiologists, American Journal of Roentgenology. (2019) 213, no. 5, W194–W210, 10.2214/AJR.19.21225. 31414888

[4] Corlu L. , Rioux-Leclercq N. , Ganard M. , Decaux O. , Houot R. , and Vigneau C. , Renal Dysfunction in Patients With Direct Infiltration by B-Cell Lymphoma, Kidney International Reports. (2019) 4, no. 5, 688–697, 10.1016/j.ekir.2019.02.008. PMC6506703 31080924

[5] Ganeshan D. , Iyer R. , Devine C. , Bhosale P. , and Paulson E. , Imaging of Primary and Secondary Renal Lymphoma, American Journal of Roentgenology. (2013) 201, no. 5, W712–W719, 10.2214/AJR.13.10669. 24147501

[6] Rosner M. H. and Perazella M. A. , Acute Kidney Injury in the Patient With Cancer, Kidney Res Clin Pract. (2019) 38, no. 3, 295–308, 10.23876/j.krcp.19.042. PMC6727896 31284363

[7] Grogg K. L. , Miller R. F. , and Dogan A. , HIV Infection and Lymphoma, Journal of Clinical Pathology. (2007) 60, no. 12, 1365–1372, 10.1136/jcp.2007.051953. PMC2095580 18042692

[8] Rivera F. B. , Ansay M. F. M. , Golbin J. M. et al., HIV-Associated Nephropathy in 2022, Glomerular Diseases. (2023) 3, no. 1, 1–11, 10.1159/000526868. PMC9936764 36816427

[9] Sellin L. , Friedl C. , Klein G. , Waldherr R. , Rump L. C. , and Weiner S. M. , Acute Renal Failure due to a Malignant Lymphoma Infiltration Uncovered by Renal Biopsy, Nephrology Dialysis Transplantation. (2004) 19, no. 10, 2657–2660, 10.1093/ndt/gfh201. 15388825

[10] Monfared A. , Orangpoor R. , Fakheri T. , and Falahatkar S. , Acute Renal Failure and Bilateral Kidney Infiltration as the First Presentation of Non-Hodgkin Lymphoma, Iran J Kidney Dis. (2009) 3, no. 1, 50–53. 19377260

[11] Cohen L. J. , Rennke H. G. , Laubach J. P. , and Humphreys B. D. , The Spectrum of Kidney Involvement in Lymphoma: A Case Report and Review of the Literature, American Journal of Kidney Diseases. (2010) 56, no. 6, 1191–1196, 10.1053/j.ajkd.2010.07.009. PMC3222147 20843590

[12] Prabhakar D. , Gupta K. L. , Gochhait D. et al., Rapidly Progressive Renal Failure in a Patient With Extranodal Non-Hodgkin’s Lymphoma, Indian Journal of Nephrology. (2015) 25, no. 1, 43–45, 10.4103/0971-4065.140723. PMC4323911 25684871

[13] Mitome T. , Furuya K. , Imano M. et al., Bilateral Renal Lymphoma: Rapid Recovery From an Acute Kidney Injury After Open Renal Biopsy, Clinical Case Reports. (2016) 4, no. 1, 5–8, 10.1002/ccr3.401. PMC4706393 26783426

[14] Rajakumar V. , Balaraman V. , Balasubramaniam R. , Shankar S. , Ganesan T. S. , and Kurien A. A. , Lymphoblastic Lymphoma Presenting as Bilateral Renal Enlargement Diagnosed by Percutaneous Kidney Biopsy: Report of Three Cases, Indian Journal of Nephrology. (2016) 26, no. 4, 298–301, 10.4103/0971-4065.179368. PMC4964694 27512306

[15] Saadat S. , Mahmud S. N. , and Qureshi A. , Renal Infiltration by Diffuse Large B-Cell Lymphoma as a Rare Cause of Fanconi’s Syndrome: A Case Report, Cureus. (2016) 8, no. 11, 10.7759/cureus.904. PMC5208554 28070473

[16] Okubo T. , Hirashio S. , Shimizu M. , Kuroda Y. , Doi S. , and Masaki T. , Diffuse Large B-Cell Lymphoma Presenting as Bilateral Renal Infiltration Leading to Acute Kidney Injury, CEN Case Reports. (2017) 6, no. 2, 140–147, 10.1007/s13730-017-0261-6. PMC5694399 28593485

[17] Aminde J. A. , Adze N. E. , Dedino G. A. , and Aminde L. N. , Acute Kidney Injury as Initial Presentation of Renal Lymphoma: Diagnostic and Therapeutic Challenges in a resource-limited Setting, SAGE Open Medical Case Reports. (2019) 7, 10.1177/2050313X19845251. PMC6487745 31065357

[18] Bai Y. , Zheng Y. , Zhang Q. et al., Renomegaly and Acute Kidney Injury as Primary Manifestations of Non-Hodgkin’s Lymphoma: a Report of Three Cases, Diagnostic Pathology. (2023) 18, no. 1, 10.1186/s13000-023-01408-7. PMC10704622 38066507

[19] Bertacchi M. , Vasey L. , Wilhelm-Bals A. , Rougemont A. L. , Baleydier F. , and Parvex P. , Primary Renal Diffuse Large B-Cell Lymphoma Presenting as New-Onset Kidney Failure, Pediatric Nephrology. (2025) 40, no. 10, 3181–3186, 10.1007/s00467-025-06833-y. PMC12401750 40493263

[20] Cetin T. E. , Yahyaoglu R. G. , Saribiyik I. , Ertunc O. , Akcay O. F. , and Erten Y. , A Case of Renal Diffuse Large B-Cell Lymphoma Concurrent With Disseminated Muscle Involvement, BMC Nephrology. (2025) 26, no. 1, 10.1186/s12882-025-04503-7. PMC12506262 41057983

[21] Darwish N. and Lightle A. , Acute Renal Failure as the Initial Presentation of T-Lymphoblastic Lymphoma: A Case Report, Clinical Nephrology—Case Studies. (2025) 13, no. 1, 103–108, 10.5414/CNCS111766. PMC12723600 41445717

[22] Panel on Antiretroviral Guidelines for Adults and Adolescents , Guidelines for the Use of Antiretroviral Agents in Adults and Adolescents With HIV, Department of Health and Human Services, https://clinicalinfo.hiv.gov/en/guidelines/adult-and-adolescent-arv.

[23] Barta S. K. , Xue X. , Wang D. et al., Treatment Factors Affecting Outcomes in HIV-Associated Non-Hodgkin Lymphomas: A Pooled Analysis of 1546 Patients, Blood. (2013) 122, no. 19, 3251–3262, 10.1182/blood-2013-04-498964. PMC3821722 24014242

